# Engineering *Saccharomyces cerevisiae* for the production and secretion of Affibody molecules

**DOI:** 10.1186/s12934-022-01761-0

**Published:** 2022-03-09

**Authors:** Veronica Gast, Anna Sandegren, Finn Dunås, Siri Ekblad, Rezan Güler, Staffan Thorén, Marta Tous Mohedano, Mikael Molin, Martin K. M. Engqvist, Verena Siewers

**Affiliations:** 1 Department of Biology and Biological Engineering, Chalmers University of Technology, Gothenburg, Sweden; 2 Novo Nordisk Foundation Center for Biosustainability, Chalmers University of Technology, Gothenburg, Sweden; 3 Affibody AB, Stockholm, Sweden

**Keywords:** Recombinant protein production, Heterologous protein production, Secretion, Proteases, Pep4, Prc1, Prb1, Fed-batch, *Saccharomyces cerevisiae*, Yeast

## Abstract

**Background:**

Affibody molecules are synthetic peptides with a variety of therapeutic and diagnostic applications. To date, Affibody molecules have mainly been produced by the bacterial production host *Escherichia coli.* There is an interest in exploring alternative production hosts to identify potential improvements in terms of yield, ease of production and purification advantages. In this study, we evaluated the feasibility of *Saccharomyces cerevisiae* as a production chassis for this group of proteins.

**Results:**

We examined the production of three different Affibody molecules in *S. cerevisiae* and found that these Affibody molecules were partially degraded. An albumin-binding domain, which may be attached to the Affibody molecules to increase their half-life, was identified to be a substrate for several *S. cerevisiae* proteases. We tested the removal of three vacuolar proteases, proteinase A, proteinase B and carboxypeptidase Y. Removal of one of these, proteinase A, resulted in intact secretion of one of the targeted Affibody molecules. Removal of either or both of the two additional proteases, carboxypeptidase Y and proteinase B, resulted in intact secretion of the two remaining Affibody molecules. The produced Affibody molecules were verified to bind their target, human HER3, as potently as the corresponding molecules produced in *E. coli* in an in vitro surface-plasmon resonance binding assay. Finally, we performed a fed-batch fermentation with one of the engineered protease-deficient *S. cerevisiae* strains and achieved a protein titer of 530 mg Affibody molecule/L.

**Conclusion:**

This study shows that engineered *S. cerevisiae* has a great potential as a production host for recombinant Affibody molecules, reaching a high titer, and for proteins where endotoxin removal could be challenging, the use of *S. cerevisiae* obviates the need for endotoxin removal from protein produced in *E. coli*.

**Supplementary Information:**

The online version contains supplementary material available at 10.1186/s12934-022-01761-0.

## Background

Affibody molecules are synthetic peptides that can be designed to bind with high affinity to other proteins. The part of the protein exhibiting binding properties originates from the B domain of the immunoglobulin-binding region of the staphylococcal protein A (SpA) [1]. Nilsson et al. isolated the B domain of SpA and increased its stability, which led to the creation of the synthetic Z domain [2]. By randomizing 13 surface-exposed residues the specificity and affinity of the Z domain can be altered. Over the years, several Z domain variants have been generated with affinity to diagnostically relevant substrates, such as cancer markers [3–5]. A specific example of this is HER3. Elevated HER3 expression is associated with malignant cancer in ovarian, prostate, gastric, bladder, lung, melanoma and colorectal tissue [6]. Current applications for Affibody molecules are mostly within diagnostics and therapeutics where the main advantage of Affibody molecules compared to alternatives, like antibodies, is their small size which allows them to penetrate tissue more easily [7, 8].

Affibody molecules can be designed in a range of configurations, either containing solely Z-domains or Z-domains together with other peptide sequences. Affibody molecules composed of only the Z domain exhibit a relatively short half-life in the human body. Due to their small size, 6.7 kDa for a single Z domain, Affibody molecules are taken up by the kidneys and degraded [9]. To prolong the half-life of the Affibody molecules an albumin binding domain (ABD) can be added. The ABD originates from the GA3 module of streptococcal protein G and binds to serum albumin. Serum albumin, with a size of 67 kDa, is above the glomerular filtration barrier of the kidneys and adding ABD to Affibody molecules thus increases both their half-life and efficacy [5, 10]. Different configurations of the Z domain and the ABD domain, like the presence of multiple Z domains or an alternative localization of the ABD within the molecule, have shown to be effective in a range applications [7, 10, 11].

Currently, Affibody molecules are produced in the bacterial production host *Escherichia coli*. Bacteria are popular hosts for recombinant production due to rapid growth and high yields of the recombinant proteins [12]. However, recombinant protein production in *E. coli* leads to contamination with lipopolysaccharides from the bacterial cell wall, most of which exhibit endotoxin properties and can be difficult to remove during product purification [13, 14]. It is therefore of interest to explore other established and competitive production hosts like yeasts that do not have toxic components in their cell wall.

Yeasts are eukaryotic unicellular organisms, which secrete recombinant proteins and have been implemented as industrial production hosts for several pharmaceutical proteins [15, 16]. The most widely used yeast species for the industrial production of recombinant pharmaceutical proteins are *Saccharomyces cerevisiae* and *Komagataella phaffi* [16]. *K. phaffi* has been used as production host of a fusion construct of human serum albumin and a HER2 Affibody molecule; however, *S. cerevisiae* has not been tested as an Affibody molecule production host yet [17]. *S. cerevisiae* on the other hand is particularly known for its industrial production of insulin, human serum albumin, and hepatitis vaccines [18].

In this study, we tested an engineered *S. cerevisiae* strain for the production and secretion of three Affibody molecules that bind to the cancer marker protein HER3 [6]. Initially, we found that *S. cerevisiae* degraded the produced Affibody molecules. We were able to identify several proteases responsible for this degradation, and upon removal of these, all three Affibody molecules were secreted in an intact state. We verified high-affinity HER3 binding by one of the secreted Affibody molecules and performed a fed-batch cultivation where a high final titer was reached thus demonstrating that *S. cerevisiae* is a competitive host for Affibody molecule production.

## Results

### The albumin-binding domain is degraded by *S. cerevisiae*

**Fig. 1:**
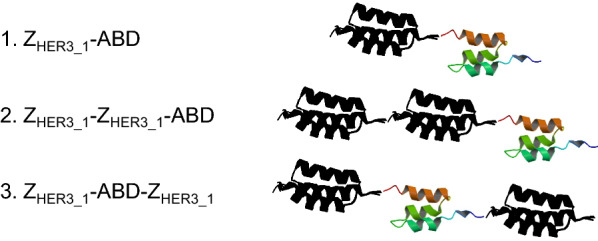
Schematic representation of domain configurations in the three Affibody molecules produced in yeast. The Z_HER3_1_ domain is the black peptide and the albumin binding domain (ABD) the colored peptide

**Fig. 2:**
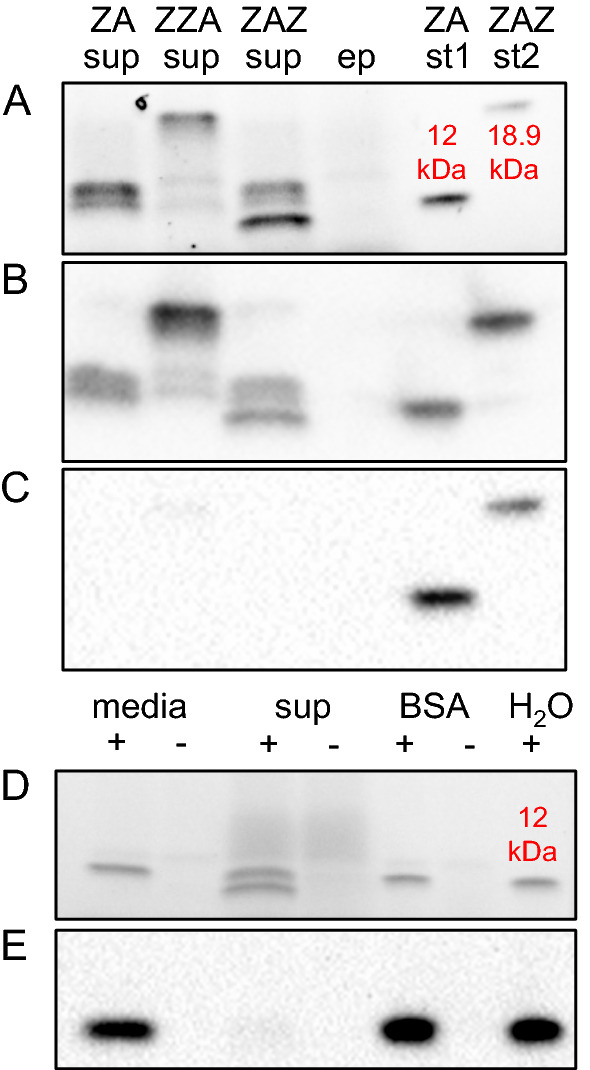
SDS-PAGE and western blot analysis of supernatant of B184k expressing different Affibody molecules. **A** SDS-PAGE of supernatant after 48 h of cultivation of B184k producing three different Affibody molecules (cropped). The lane marked with ZA sup contains supernatant of B184k expressing Z_HER3_1_-ABD, the lane marked with ZZA sup contains supernatant of B184k expressing Z_HER3_1_-Z_HER3_1_-ABD, the lane marked with ZAZ sup contains supernatant of B184k expressing Z_HER3_1_-ABD-Z_HER3_1_, the lane marked with ep contains supernatant of B184k with empty plasmid, the lane marked with ZA and st1 contains supernatant of Z_HER3_1_-ABD standard (12 kDa) dissolved in water, the lane marked with ZAZ and st2 contains supernatant of Z_HER3_1_-ABD-Z_HER3_1_ standard (18.9 kDa) dissolved in water. **B** Western blot against the Z-domain (cropped), the membrane was blotted with anti-Z-domain antibody (1:1000) followed by an anti-Mouse (1:5000) secondary antibody. **C** Western blot against the ABD (cropped), the membrane was blotted with anti-ABD antibody (3:1000) followed by an anti-Rabbit (1:5000) secondary antibody. **D** SDS-PAGE of supernatant of dissolved Z_HER3_1_-ABD in different solutions (cropped). The lane marked with media contains fresh SD2xSCAA media with (+) and without (−) Z_HER3_1_-ABD standard added, The lane marked with sup contains spent supernatant of a 24-h old culture of B184k in SD2xSCAA medium with (+) and without (−) Z_HER3_1_-ABD standard added, the lane marked with BSA contains water with 0.1% BSA with (+) and without (−) Z_HER3_1_-ABD standard added and the lane marked with H_2_O, + contains Z_HER3_1_-ABD standard in water. **E** Western blot against the ABD (cropped), the membrane was blotted with anti-ABD antibody (3:1000) followed by an anti-Rabbit (1:5000) secondary antibody

We first tested whether Affibody molecules could be produced and secreted by *S. cerevisiae.* We included three different Affibody molecules in this study. These three molecules have different configurations of either one or two Z_HER3_1_ domains, the Z domain variant that binds to HER3, and one albumin binding domain (ABD) (Fig. 1). *S. cerevisiae* strain B184k, previously evolved for high levels of protein secretion, was used as the host [19, 20]. Genes encoding the three different Affibody molecules shown in Fig. 1 were cloned into the backbone of a CPOT plasmid, thus generating the three plasmids pNatZACPOT (Z_HER3_1_-ABD), pNatZZACPOT (Z_HER3_1_-Z_HER3_1_-ABD), and pNatZAZCPOT (Z_HER3_1_-ABD-Z_HER3_1_) [21]. CPOT is a recombinant protein expression plasmid for use in *S. cerevisiae tpi1*Δ strains*.* The CPOT plasmid contains the *POT1* gene encoding triose phosphate isomerase from *Schizosaccharomyces pombe*, which partially complements the removal of the *TPI1* gene and restores the ability to grow on glucose as a carbon source. The partial complementation will lead to a high abundancy of the plasmid in the cell, which is combined with the expression regulation of the recombinant gene by the native *TPI1* promoter and terminator [21]. The combination of the CPOT expression system and B184 has shown effective for high-level production of several recombinant proteins [19, 20]. The plasmids pNatZACPOT, pNatZZACPOT and pNatZAZCPOT were used to transform B184k and the positive transformants were grown for 48 h in liquid SD2xSCAA media [22]. The supernatant was analyzed by a reducing SDS-PAGE followed by western blot using antibodies against both the ABD and the Z domain (Fig. 2A–C). Although all three Affibody molecules were secreted the bands did not appear with the expected sizes on the blot. We expected a 12 kDa band for Z_HER3_1_-ABD and 18.9 kDa for Z_HER3_1_-ABD-Z_HER3_1_ and Z_HER3_1_-Z_HER3_1_-ABD. The supernatant derived from the Z_HER3_1_-ABD-Z_HER3_1_ expressing strain showed three separate smaller bands on the anti-Z-domain blot with sizes compared to the Z_HER3_1_-ABD standard around 12 kDa instead of one intact band seen in the Affibody standard around 18.9 kDa (Fig. 2B). The supernatant of the Z_HER3_1_-ABD expressing strain showed two bands instead of one on the anti-Z-domain blot, which appeared around the expected size of 12 kDa (Fig. 2B). The anti-Z-domain blot for Z_HER3_1_-Z_HER3_1_-ABD showed an apparently intact band around the correct size of 18.9 kDa (Fig. 2B). Finally, the ABD domain showed none or little signal on the anti-ABD blot for any of the Affibody molecules (Fig. 2C). The absence of bands on the anti-ABD blots indicated an absence of intact ABD in the secreted Affibody molecules.

We hypothesized that the secreted molecules were degraded by *S. cerevisiae* proteases leading to fragmentation (Fig. 2B and C). We also checked if the presence of BSA might have an influence. Since the albumin-binding domain binds to serum albumin [5], and one of the components of the SD2xSCAA media that was used for cultivation was bovine serum albumin (BSA), we wanted to exclude any BSA-related interference. We tested our hypotheses by incubating one Affibody molecule standard for the Z_HER3_1_-ABD in four different solutions. The solutions were fresh sterile medium, spent supernatant, demineralized water with 0.1% BSA and demineralized water. The spent supernatant is the supernatant of cultures in which yeast cells had been cultivated and were removed by centrifugation. If the absence of the intact ABD was due to proteolytic activity, we expected to see degradation by incubating the Affibody molecule standard in the spent supernatant. The spent supernatant for this experiment was derived from a 24-h culture of B184k producing α-amylase. After the incubation, the proteins were separated by reducing SDS-PAGE and analysed by western blotting against the ABD (Fig. 2D and E). The western blot shows intact bands for the fresh sterile medium, demineralized water with 0.1% BSA, and demineralized water. For the spent supernatant, the stained SDS-PAGE shows two bands instead of one and there is no band visible on the western blot against the ABD, which indicates degradation of the Affibody molecule in the spent supernatant.

### Affibody molecules are degraded by aspartyl protease(s)

Proteolytic activity can drastically decrease recombinant protein yield; however, this can often at least partially be prohibited by the identification and removal of the responsible proteases [23]. First, we wanted to check if the degradation was a characteristic of the engineered yeast strain B184k, since a potential solution for the degradation would be to switch to another strain. We checked the supernatant of the parental strain of B184k, AAC [19]. The influence of expression of a recombinant protein on the degradation was also tested by the expression of α-amylase by AAC. Both B184k and AAC containing either pNatAmyCPOT or an empty CPOT plasmid were cultivated. The Affibody standard of Z_HER3_1_-ABD-Z_HER3_1_ was incubated in spent medium from those cultivations, and the mixture with the proteins was separated by reducing SDS-PAGE (Additional file 1: Fig. S1). We included supernatant after 24, 48, 72, and 96 h of cultivation and tested the presence of a protease inhibitor cocktail to confirm that the degradation was due to proteases. Degradation of the Affibody molecules was also observed in medium spent by the strain AAC (Additional file 1: Fig. S1). In the supernatant containing protease inhibitor cocktail, we did not observe degradation, confirming that the fragmentation of the Affibody molecules was a result of proteolytic activity. We observed a similar degradation pattern as for B184k. Degradation, however, seemed slightly elevated in supernatant derived from strains with recombinant α-amylase production, which could potentially indicate an induction of protease expression or activity upon recombinant protein production. Since the degradation was observed also in the parental strain of B184k we concluded that the degradation is not a characteristic of B184k. Therefore, we continued with B184k as the production host.

**Fig. 3:**
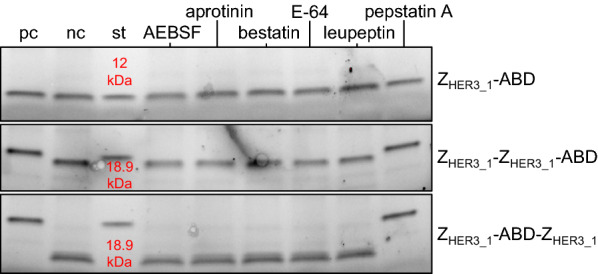
Protease inhibitor assay with spent supernatant of B184 and Affibody molecule standards. SDS-PAGE of spent supernatant with the indicated Affibody molecules and addition of different protease inhibitors (cropped). The supernatant is derived from a cultivation of B184k producing α-amylase grown for 24 h at 30 °C at 220 rpm. The Affibody molecule standards were incubated overnight at 30 °C at 220 rpm. The lane marked with pc contains the positive control with spent supernatant with the respective Affibody molecule and the complete protease inhibitor cocktail, the lane marked with nc contains the negative control with spent supernatant with the respective Affibody molecule, the lane marked with st contains the standard of the respective Affibody molecule in water, the lane marked with AEBSF contains spent supernatant with the respective Affibody molecule with 4-(2-aminoethyl) benzenesulfonyl fluoride hydrochloride 1 mM, the lane marked with aprotinin contains spent supernatant with the respective Affibody molecule with aprotinin 6.5 μg/mL, the lane marked with bestatin contains spent supernatant with the respective Affibody molecule with bestatin hydrochloride 50 μM, the lane marked with E-64 contains spent supernatant with the respective Affibody molecule with E-64 15 μM, the lane marked with leupeptin contains spent supernatant with the respective Affibody molecule with leupeptin 20 μM and the lane marked with pepstatin A contains spent supernatant with the respective Affibody molecule with pepstatin A 10 μM

The next step was to identify the proteases responsible for the degradation. Protease inhibitors mostly block the activity of a specific class of proteases. Therefore, a mixture of different protease inhibitors is combined in protease inhibitor cocktails to ensure inhibition of all sort of proteases. Since in the experiment with AAC (Additional file 1: Fig. S1) a protease inhibitor cocktail showed effective against the degradation we tested the isolated inhibitors from that specific cocktail. The spent medium of B184k expressing α-amylase was incubated overnight with Affibody molecule standards and the individual components of the protease inhibitor cocktail. The mixture was subsequently analyzed by a reducing SDS-PAGE. The results of the SDS-PAGE show that the Affibody molecules were degraded in the presence of AEBSF, aprotinin, bestatin, E-64 or leupeptin (Fig. 3). Only incubation with pepstatin A prohibited degradation and resulted in Affibody molecules with the correct size (Fig. 3). Both the Affibody molecules Z_HER3_1_-Z_HER3_1_-ABD and Z_HER3_1_-ABD-Z_HER3_1_ clearly showed the absence of proteolytic processing on the SDS-PAGE in the presence of pepstatin A. For Z_HER3_1_-ABD, the difference was less explicit which could be a result of the small size difference between the intact and degraded molecule. Pepstatin A inhibits aspartyl proteases, indicating that the protease(s) responsible for Affibody molecule processing are of this type.

### The removal of several proteases results in the secretion of intact Affibody molecules

**Fig. 4:**
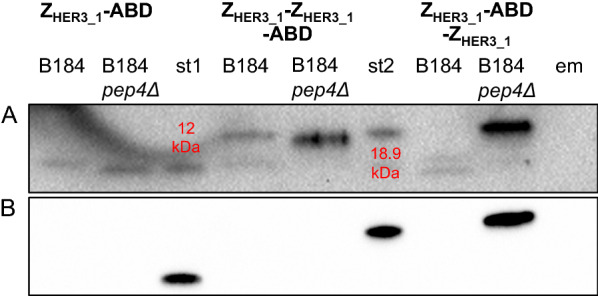
Western blot analysis of the Affibody molecules produced in B184 and B184 *pep4Δ*. Western blot against the Z-domain (cropped) (**A**) and against ABD (cropped) (**B**) in the supernatant of B184 and B184 *pep4Δ* expressing the three Affibody molecule genes after 96 h of cultivation. For the western blot against the Z-domain, the membrane was blotted with anti-Z-domain antibody (1:1000) followed by an anti-Mouse (1:5000) secondary antibody. For the Western blot against ABD, the membrane was blotted with anti-ABD antibody (3:1000) followed by an anti-Rabbit (1:5000) secondary antibody. The lane marked with st1 contains the standard of Z_HER3_1_-ABD (10 mg/L) in water, the lane marked with st2 contains the standard of Z_HER3_1_-ABD-Z_HER3_1_ (10 mg/L) in water and the lane marked with em contains empty SD2xSCAA media

One of the major proteases in *S. cerevisiae* and a proven target for improving recombinant protein production yield is a vacuolar aspartyl protease, proteinase A [23–25]. We removed the *PEP4* gene encoding proteinase A in B184 using CRISPR/Cas9-based gene deletion and expressed all three Affibody molecules in B184 *pep4*Δ. Z_HER3_1_-ABD-Z_HER3_1_ produced by this strain was intact and showed a signal on western blots using antibodies against either the Z-domain or ABD (Fig. 4), indicating that proteinase A was indeed responsible for its cleavage in B184. In contrast, for Z_HER3_1_-ABD and Z_HER3_1_-Z_HER3_1_-ABD, the western blot against the Z-domain showed a small shift of the bands upon removal of *PEP4* but there was no band visible on the blot against the ABD (Fig. 4A). We suspected that at least one other protease was involved in cleaving the ABD domain of Z_HER3_1_-ABD and Z_HER3_1_-Z_HER3_1_-ABD in B184 *pep4*Δ.

**Fig. 5:**
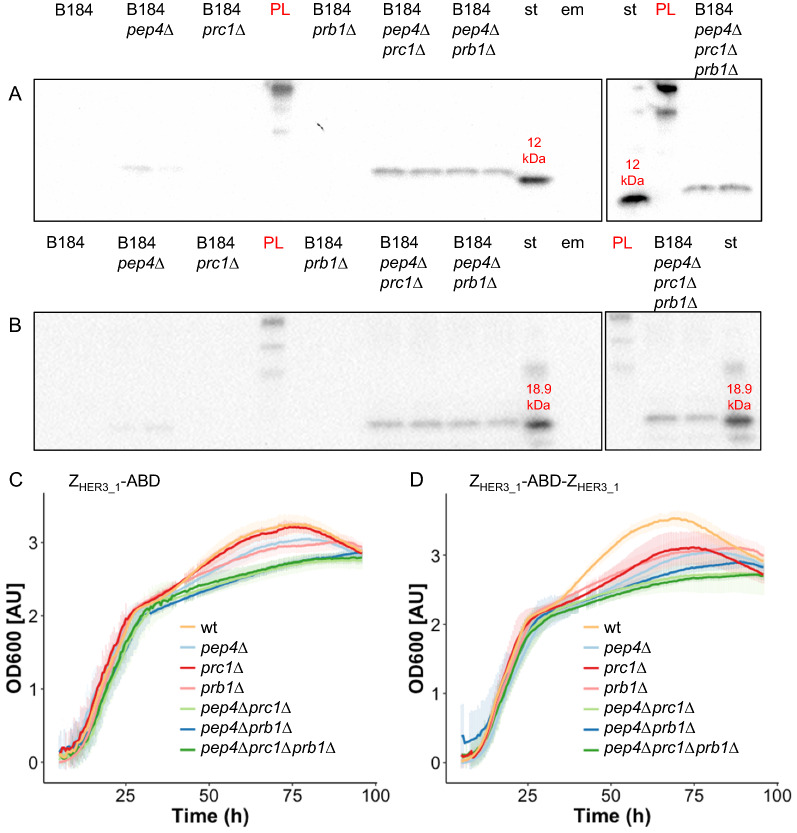
Affibody molecule production and growth of B184 carrying deletions of three protease genes. **A**, **B** Western blot analysis against the ABD (cropped). The samples are from a 48-h cultivation of B184, B184 *pep4Δ,* B184 *prc1Δ,* B184 *prb1Δ,* B184 *pep4Δprc1Δ,* B184 *pep4Δprb1Δ* and B184 *pep4Δprc1Δprb1Δ* expressing Z_HER3_1_-ABD (**A**) or Z_HER3_1_-Z_HER3_1_-ABD (**B**). In the Western blot against ABD, the membrane was blotted with anti-ABD antibody (3:1000) followed by an anti-Rabbit (1:5000) secondary antibody. The lane marked with st contains the standard of the respective Affibody molecule, the lane marked with em contains empty SD2xSCAA medium and the lane marked with PL contains the protein ladder. **D** OD_600_ development during 96 h of growth in SD2xSCAA media of B184, B184 *pep4Δ,* B184 *prc1Δ,* B184 *prb1Δ,* B184 *pep4Δprc1Δ,* B184 *pep4Δprb1Δ* and B184 *pep4Δprc1Δprb1Δ* expressing Z_HER3_1_-ABD (**C**) or Z_HER3_1_-Z_HER3_1_-ABD (**D**). The graphs show averaged data based on biological triplicates each assessed in technical triplicate analysis. The lighter bars show the standard deviation. The first 5 h were excluded from the graphs due to noise

We therefore removed two additional proteases in B184 *pep4*Δ in the hope of producing intact Z_HER3_1_-ABD and Z_HER3_1_-Z_HER3_1_-ABD, namely carboxypeptidase Y and proteinase B. Carboxypeptidase Y is a serine-based exopeptidase active in the vacuole and proteinase B, a serine-based endopeptidase also located to the vacuole. Proteinase B, together with proteinase A, plays an essential role in the activation of several vacuolar proteases including itself and carboxypeptidase Y. The *PRC1* gene encoding carboxypeptidase Y and the *PRB1* gene encoding proteinase B were removed in B184 and B184 *pep4*Δ (*pep4*Δ*prc1*Δ and *pep4*Δ*prb1*Δ) and a triple deletion strain (*pep4*Δ*prc1*Δ*prb1*Δ) was constructed. The single, the two double and the triple deletion strains were grown for 48 h in an aerated 24-well plate and the supernatant in which triple deletion strains expressing the Affibody molecules were grown was analyzed for the presence of intact Affibody molecules. In the B184 *pep4*Δ*prc1*Δ strain we observed intact Z_HER3_1_-ABD and Z_HER3_1_-Z_HER3_1_-ABD (Fig. 5A and B). Interestingly, the removal of solely *PEP4* during cultivation for 48 h instead of 96 h, resulted in very weak bands for the ABD for both Z_HER3_1_-ABD and Z_HER3_1_-Z_HER3_1-_ABD on the blot against the ABD. This indicates that the degradation in B184 *pep4*Δ could primarily occur at later cultivation stages. The single removal of either *PRC1* or *PRB1* did not result in a band on the western blot using antibody against the ABD. However, the combination of *pep4*Δ (proteinase A) with either *prb1*Δ (proteinase B) or *prc1*Δ (carboxypeptidase Y) resulted in the production of intact Z_HER3_1_-ABD as well as intact Z_HER3_1_-ABD-Z_HER3_1_. The deletion of all three proteases (*pep4*Δ*prc1*Δ*prb1*Δ) resulted in titers comparable to the two double deletion strains (Fig. 5A and B).

Proteases fulfill a crucial role in the degradation of macromolecules in the vacuole and have even been reported to benefit aging [26, 27]. Therefore, the removal of one to three major proteases can have an impact on vacuolar homeostasis and cellular growth. We monitored the growth profiles of the protease-deficient strains in aerated 96-well plates. The protease deficiency led to a slight reduction of final OD_600_ in the later stages of the growth, from 50 to 96 h, but overall growth of the deletion strains, even the triple deletion strain, was similar to B184 with the proteases intact. This was observed for both Z_HER3_1_-ABD and Z_HER3_1_-Z_HER3_1_-ABD (Fig. 5C and D).

**Fig. 6:**
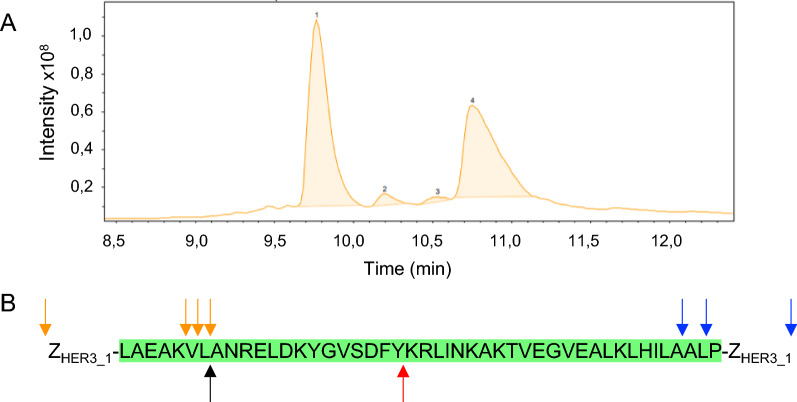
Fragmentation of Z_HER3_1_-ABD-Z_HER3_1_ based on LC–MS analysis. The TIC chromatogram Z_HER3_1_-ABD-Z_HER3_1_ standard was incubated in spent supernatant of B184k while producing α-amylase. **A** Total Ion chromatogram (TIC), **B** Visualization of the ABD amino acid sequence and identified cut sites. The green marked sequence indicates the ABD. The blue arrows indicate the ends of detected fragments with intact C-terminus and a cut site within the ABD and the orange arrows indicate a detected fragment with intact N-terminus and a cut site within the ABD. These fragments are based on the first peak of the TIC. The red and black arrows indicate beginning and ends of two additional detected fragments that were determined based on the fourth peak which had their C- and N-terminus within the Z_HER3_1_-ABD-Z_HER3_1_ molecule

Additionally, we were interested in identifying the cleavage site of the endopeptidase proteinase A in the ABD. It is known that proteinase A has a broad and variable activity for different ligands and seems to favor cut sites between adjacent hydrophobic residues [28, 29]. We incubated the Z_HER3_1_-ABD-Z_HER3_1_ standard in spent supernatant for 24 h and analysed the mixture with mass spectrometry. The total ion chromatogram (TIC) showed four peaks, two large peaks and two smaller ones (Fig. 6A). The peptides within the two larger peaks were analysed. In the first peak, several fragments of Z_HER3_1_-ABD-Z_HER3_1_ were identified where either the N- or C-terminus was intact and the other terminus was located within the ABD indicating a cut site of an endopeptidase (Fig. 6B). The peptides in the fourth peak were fragments which had the N-terminus in the ABD and the C-terminus in the second Z_HER3_1_ sequence (Fig. 6B). Based on these fragments it seems that proteinase A cleaves at several sites within the ABD and interestingly the C-terminal Z_HER3_1_ domain was also not intact.

### Z_HER3_1_-ABD-Z_HER3_1_ molecule produced by B184 *pep4Δ* shows similar binding kinetics compared to the molecule produced by *E. coli*

We next aimed to test whether the engineered *S. cerevisiae* strain is a competitive host for the Affibody molecule production with regards to product quality. Since the three Affibody molecules consist of the same two subunits, Z_HER3_1_ and ABD, we decided to include only one version in the subsequent studies as a proof of concept. The Z_HER3_1_-ABD-Z_HER3_1_ molecule produced and secreted by B184 *pep4*Δ was tested in a surface plasmon resonance binding assay with the substrate of Z_HER3_1_, HER3. The Z_HER3_1_-ABD-Z_HER3_1_ Affibody molecule has two binding sites for HER3; therefore, the bivalent binding kinetics were determined and presented as average value of duplicates in Table 1. The equilibrium dissociation constant of the first site has a value in the nano molar range, which indicates high affinity. The values of the Z_HER3_1_-ABD-Z_HER3_1_ molecules secreted by *E. coli* and *S. cerevisiae*, respectively, show comparable kinetics which shows that the molecules produced by *S. cerevisiae* are fully functional (Table 1, Additional file 1: Fig. S2).

**Table 1:** Bivalent binding kinetics assay parameters of Z_HER3_1_-ABD-Z_HER3_1_ produced by B184 *pep4Δ* and by the original host *E. coli*

Production host	k_a_1 (1/Ms)	k_a_2 (1/RUs)	k_d_1 (1/s)	k_d_2 (1/s)	K_D_1 (M)	K_D_2 (M)
*E. coli*	5.21 × 10^5^	1.23 × 10^–3^	1.43 × 10^–2^	2.29 × 10^–5^	2.75 × 10^–8^	1.85 × 10^–2^
*S. cerevisiae*	3.83 × 10^5^	1.71 × 10^–3^	1.79 × 10^–2^	2.76 × 10^–5^	4.68 × 10^–8^	1.62 × 10^–2^

k_a_1 = association rate constant of the first site; k_a_2 = association rate constant of the second site; k_d_1 = dissociation rate constant of the first site; k_d_2 = dissociation rate constant of the second site; K_D_1 = equilibrium dissociation constant for the first site, K_D_2 = equilibrium dissociation constant for the second site

### B184 *pep4Δ* produces a high titer of Z_HER3_1_-ABD-Z_HER3_1_ in a fed-batch bioreactor

**Fig. 7:**
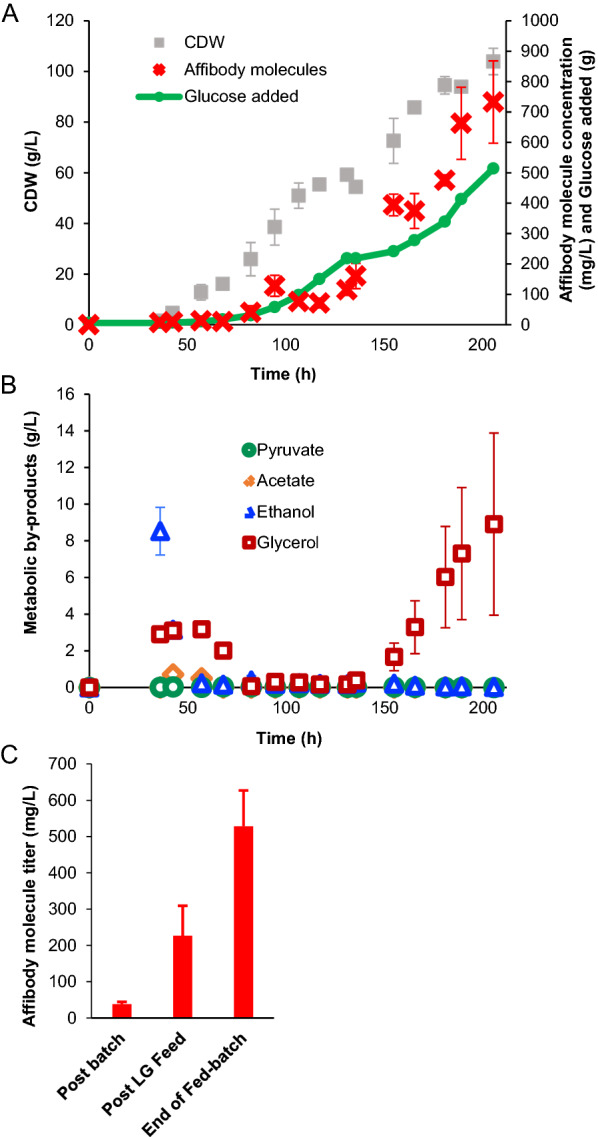
Fed-batch fermentation of B184 *pep4Δ* expressing Z_HER3_1_-ABD-Z_HER3_1_. Experimental data from a fed-batch bioreactor experiment of B184 *pep4Δ* expressing Z_HER3_1_-ABD-Z_HER3_1_. The cells were cultured in SD2xSCAA medium and condensed feed media. Details of the feed media and strategy are presented in the materials and methods section. **A** Affibody concentration, cell dry weight (CDW) and glucose addition to the bioreactors. The quantity of the Affibody molecule concentration is based on western blot analysis against the albumin binding domain. All the data are based on biological triplicates and the average and standard deviations are shown. **B** Metabolic byproduct concentrations in the supernatant during the fed-batch cultivation. All the data are based on biological triplicates and the average and standard deviations are shown. **C** Affibody titers after the batch phase, after the low-glucose feed phase and at the end of the fed-batch fermentation. This quantification was done using BLI determination for Z_HER3_1_-ABD-Z_HER3_1_. The data are based on biological triplicates and technical duplicates and the average and standard deviations are shown

For *S. cerevisiae* to be a suitable host for Affibody molecule production it must be able to produce competitive titers of Affibody molecules. So next, we wanted to assess the productivity of *S. cerevisiae* in bioreactors. We decided to use B184 *pep4*Δ producing Z_HER3_1_-ABD-Z_HER3_1_ as a proof of concept. To exclude any major impact of the deletion of *pep4*Δ on cellular growth of B184 while producing Z_HER3_1_-ABD-Z_HER3_1_ we tested B184 and B184 *pep4*Δ in a micro-cultivation experiment in an aerated 96-well plate (Additional file 1: Fig. S3). Deletion of *pep4*Δ conferred only a minor impact on growth (Additional file 1: Fig. S3). After this confirmation the productivity for Z_HER3_1_-ABD-Z_HER3_1_ of B184 *pep4*Δ was tested in a bioreactor experiment. The cultivation set-up was a batch fermentation followed by fed-batch fermentation. During the batch phase, a specific growth rate of 0.31 h^−1^ was measured (Additional file 1: Fig. S4) which is the same growth rate as for B184 carrying an intact *PEP4* gene and producing α-amylase which confirmed that B184 *pep4*Δ did not suffer from a growth impairment [20]. The feeding of a low-glucose feed into the bioreactors was started after 36 h and was switched to a high-glucose feed after 130 h for the remaining duration of the fermentation until 180 h. At the end of the fed-batch cultivation, the biomass concentration exceeded 100 g/L (Fig. 7A). We also observed an increasing trend of Affibody molecule titer with the duration of the experiment especially after the switch to the high-glucose feed (Fig. 7A). During the cultivation, minor production of byproducts was observed. Ethanol and glycerol were present in the medium after the batch phase but were rapidly consumed after the feed was started (Fig. 7B). At the end of the fed-batch, residual glycerol increased again and reached a final concentration of 10 g/L (Fig. 7B). We determined the exact quantity of Z_HER3_1_-ABD-Z_HER3_1_ at three timepoints using a BLI based method. The results showed a final titer of 530 mg/L (Fig. 7C).

## Discussion

In this study we examined *S. cerevisiae* as a host for Affibody molecule production. Currently, the molecules are mainly produced in *E. coli*. In the current study, we present engineered protease-deficient *S. cerevisiae* as a suitable alternative.

Our results show that all three Affibody molecules were produced and secreted by *S. cerevisiae* after the removal of only proteinase A or with the additional removal of proteinase B or carboxypeptidase Y. Removal of only proteinase A was sufficient for the production of intact Z_HER3_1_-ABD-Z_HER3_1_ and a small amount of both Z_HER3_1_-ABD and Z_HER3_1_-Z_HER3_1_-ABD in the case of a shorter cultivation of 48 h instead of 96 h. We suspect that a longer duration of the experiment would lead to more degradation. The additional removal of carboxypeptidase Y and/or proteinase B is required for the intact production of Z_HER3_1_-ABD and Z_HER3_1_-Z_HER3_1_-ABD, respectively. The ABD is in Z_HER3_1_-ABD-Z_HER3_1_ flanked by the two Z_HER3_1_ domains, which potentially protects the ABD from exopeptidase activity from proteases like carboxypeptidase Y. This would explain why we observed intact Z_HER3_1_-ABD and Z_HER3_1_-Z_HER3_1_-ABD only upon removal of carboxypeptidase Y in proteinase A deficient B184 *pep4*Δ. Additionally, we confirmed that the removal of carboxypeptidase Y does not further improve the production of Z_HER3_1_-ABD-Z_HER3_1_ in proteinase A-deficient B184 *pep4*Δ (Additional file 1: Fig. S5). Interestingly, we also observed production of intact Z_HER3_1_-ABD and Z_HER3_1_-Z_HER3_1_-ABD upon removal of proteinase B, which is an endopeptidase, in B184 *pep4*Δ and the additional deletion of proteinase B in B184 *pep4*Δ*prc1*Δ (lacking proteinase A and carboxypeptidase Y) or carboxypeptidase Y in B184 *pep4*Δ*prb1*Δ (lacking proteinase A and proteinase B) did not influence the titer of intact Z_HER3_1_-ABD and Z_HER3_1_-Z_HER3_1_-ABD. This observation can be explained by the fact that proteinase A, proteinase B and carboxypeptidase Y are all three vacuolar proteases that before reaching their final destination, the vacuole, pass through the secretory pathway as inactive precursors with a prepro-signalpeptide [30]. The pre-signal peptide is removed after arrival in the ER and the pro-signal peptide during trafficking to or upon arrival in the vacuole [30]. Proteases can have multiple pro-signal peptides, which prohibits the proteases to reach their active form. Proteinase A and B are responsible for the removal of pro-signal peptide(s) on other vacuolar proteases and are therefore a central part of the activation cascade of vacuolar proteases.

For maturation of our deletion targets proteinase A, proteinase B and carboxypeptidase Y, activity of both proteinase A and B is required to reach their activate form. The maturation of proteinase A starts with an auto activation step to the pseudo form upon entering the vacuole, which is followed by removal of a second pro-signal peptide by proteinase B [31, 32]. The intermediate form of the protease with one pro-signal peptide and one already removed is called the pseudo-form. The exact activation trigger for the proteases remains unknown but has been linked to the acidic pH in the vacuole [33, 34]. Proteinase B has two pro-peptides, one at the N-terminus and another at the C-terminus. The removal of the N-terminal pro-peptide is autocatalytic and occurs in the Golgi apparatus, and the C-terminal pro-peptide is removed by proteinase A [35]. Carboxypeptidase Y has an N-terminal pro-peptide which is partly removed by proteinase A and the other part by proteinase B upon arrival in the vacuole.

As explained here, proteinase B catalyzes the activation of carboxypeptidase Y and based on our results we expect that proteinase B fulfills a role in the activation of proteinase A and carboxypeptidase Y rather than to recognize Affibody molecules as a substrate. We expect, based on our results, that in a *pep4*Δ*prb1*Δ mutant carboxypeptidase Y remains in an inactive pro-form, which limits the capacity of carboxypeptidase Y to degrade the ABD [36]. We, however, observe digestion of the ABD by strain B184 *pep4*Δ. It has been shown that in a *PEP4* deficient *S. cerevisiae* strain pro-proteases of both carboxypeptidase Y and proteinase B accumulate, alongside the reduction of their activity [37]. The combination of deleting proteinase A and proteinase B for improved recombinant protein production has been reported for several microbial hosts, including *S. cerevisiae* [25, 38, 39]. Removal of solely *PEP4* has however also proven effective for production of several proteins [25, 41, 42].

The proteases travel through the secretory pathway to reach their destination, the vacuole. The initiation of the activation should start in the proximity of the vacuole or upon transport into this organelle [40]. Proteinase A and B are trafficked directly from the Golgi apparatus to the vacuole, but carboxypeptidase Y travels through a multivesicular body intermediate [26]. Even though the proteases are targeted for the vacuole, residual secretion has been reported and linked to the glucose concentration in the media. In the experiments performed in this study 2% glucose concentration in the media was used, a concentration that previously was shown to result in extracellular activity of proteinase A [34]. This supports the observations of proteinase A activity in the spent supernatant.

Another interesting aspect is the propensity of the proteases for cleavage of ABD. Since potentially even pro-Prc1 and pseudo-Prc1 seem to digest ABD, it could well be that even partly active yeast proteases retain a high propensity for cleaving the ABD as a defense mechanism against bacteria. Proteolytic activity targeting the ABD was previously found in bacterial hosts [43].

The mass spectrometry performed for the analysis of Z_HER3_1_-ABD_Z_HER3_1_ showed that the molecular weight of the molecule produced by *S. cerevisiae* consisted of an extra 700 Da compared to the molecule produced by *E. coli*. This is visible on several SDS-PAGEs and western blots (Figs. 4, 5, Additional file 1: Figs. S1 and S5). In the construct expressed by *S. cerevisiae*, a Kex2 recognition site is located behind the α-leader sequence and is processed in the ER. Between the Kex2 site and the start of the protein, a linker peptide with the amino acid sequence Glu-Glu-Gly-Glu-Gly-Ser-Met was inserted, which exhibits a molecular weight of 737 Da and thus explains the additional mass of the produced Z_HER3_1_-ABD-Z_HER3_1_. Since the binding kinetics indicate no interference of this additional peptide, we did not include the removal of the linker peptide in the scope of this study, but for future applications its removal may be desirable.

As a final experiment to show the potential of *S. cerevisiae* as interesting production host for Affibody molecules, B184 *pep4*Δ producing Z_HER3_1_-ABD-Z_HER3_1_ was grown in fed-batch bioreactors. During the fed-batch cultures, buildup of residual glycerol was observed. The quantity of glycerol accumulating showed high variation between the reactors. Glycerol is a by-product of fermentation to reoxidize NADH to counteract redox imbalances. However, we do not expect this to be the cause of the buildup [44]. During later stages of the fed-batch, the feed was controlled by the dissolved oxygen in the reactor leading to pulse feed to ensure respiratory growth. Besides, in the case of alcoholic fermentation, there should be buildup of ethanol as well [45]. Therefore, the oxygen shortage does not seem to be the source of the potential redox imbalance. Besides redox imbalance, another explanation could be the expression platform used. *TPI1* deficiency, which is the deficiency complemented by the CPOT plasmid, is known to result in the accumulation of high levels of glycerol [46]. During the later stages of the experiment, this partial complementation could become limiting leading to the accumulation of excess glycerol. Nevertheless, high biomass concentration and Affibody molecule titer were achieved during the fermentation. The titer that was reached was 0.530 g/L. For *E. coli* expression titers for different recombinant proteins are reported in the range from 0.25 g/L to 8.5 g/L [47–49]. The titer of Z_HER3_1_-ABD-Z_HER3_1_ is within this range and can be the starting point for improvement by further engineering *S. cerevisiae* or by optimizing the cultivation method in a sequential study.

The results of this study indicate that *S. cerevisiae* shows potential as a host for Affibody molecule production. Here, we tested only three different Affibody molecules, and for the future it would be interesting to expand the selection of Affibody molecules and potentially test more complex Affibody molecule based proteins like AffiMabs [7]. Secondly, our experiments show that the removal of proteases can be a powerful approach to increase productivity of heterologous proteins in *S. cerevisiae.*

## Materials and methods

### Strains and plasmids

Two previously constructed *S. cerevisiae* strains were used in this study, CEN.PK 530.1C [*MATα URA3 HIS3 LAU2 TRP1 SUC2 MAL2-8*^*c*^* tpi1(41-707)]* (AAC) and B184k. In a previous study, AAC was evolved for improved protein production by UV-mutagenesis which led to the construction of strain B184k [19]. B184k was shown to be an effective host for the production of several recombinant proteins in combination with the CPOT plasmid [19]. The CPOT plasmid contains the *Schizosaccharomyces pombe* gene *POT1*, which partially complements the *TPI1* deficiency. The recombinant protein expression cassette contains the native *TPI1* promoter and terminator and an α-leader sequence*.* The empty CPOT is a CPOT plasmid without the recombinant protein gene. The previously constructed pNatAmyCPOT was cut by restriction digest with enzymes XhoI and KpnI to remove only the α-amylase gene. Genes for Z_HER3_1_-ABD, Z_HER3_1_-Z_HER3_1_-ABD and Z_HER3_1_-ABD-Z_HER3_1_ were synthesized by GenScript. The Affibody molecule genes were codon optimized for expression in *S. cerevisiae* but without repetitive DNA sequences to reduce the risk of homologous recombination within the ORFs. The three Affibody molecule genes were amplified with homologous overhangs of the α-leader and the CPOT backbone using primers presented in Additional file 1: Table S1. The backbone and Affibody molecule genes were assembled by Gibson assembly and the final constructs verified by sequencing using primer #7 that binds in the α-leader sequence. All primers for the construction of the plasmids can be found in Additional file 1: Table S1. After the plasmid construction, the newly assembled CPOT plasmids, pNatZACPOT, pNatZZACPOT and pNatZAZCPOT were used to transform AAC and B184k. B184k still contained a kanamycin resistance marker in the *TPI1* gene. In AAC, the marker had been removed previously. The marker has two flanking *loxP* sites. pSH66 from the Euroscarf deletion marker set was used to remove the kanamycin resistance marker [50]. pSH66 contains an expression cassette for Cre recombinase under control of the *GAL1* promoter and a nourseothricin resistance gene. After the strains had been transformed with the pSH66 plasmid with selection for nourseotricin, the positive transformants were streaked out on solid media with YPGal with nourseothricin to activate Cre recombinase expression. Removal of the kanamycin marker was confirmed by absence of growth on solid media with G418. *PEP4*, *PRC1* and *PRB1* were deleted by using plasmids pECAS9-gRNA-kanMX-tPEP4, pECAS9-gRNA-kanMX-tPRC1, pECAS9-gRNA-kanMX-tPRB1, which contain both a *cas9* gene and a gRNA expression cassette [51]. The plasmids pECAS9-gRNA-kanMX-tPEP4*,* pECAS9-gRNA-kanMX-tPRC1 and pECAS9-gRNA-kanMX-tPRB1 were constructed using pECAS9-gRNA-kanMX-tHFD1 as template [51]. First, the backbone of pECAS9-gRNA-kanMX was obtained by linearizing pECAS9-gRNA-kanMX-tHFD1 by digestion with MunI and EcoRI. The ‘left’ fragment was constructed with primer #14 in combination with either #9 (*PEP4*), #11 (*PRC1*) or #13 (*PRB1*) and the ‘right’ fragment was constructed with primer #15 in combination with either #8 (*PEP4*), #10 (*PRC1*) or #12 (*PRB1*). The correct assembly of the plasmids was confirmed by sequencing using primer #16. The genes were deleted in B184 pNatZACPOT, B184 pNatZZACPOT and B184 pNatZAZCPOT. The genomic deletions were verified using forward primers #17 and #18 with reverse primer #19 for *PEP4*, forward primers #20, #21 and reverse primer #22 for *PRC1,* and forward primers #23, #24 and reverse primer #25 for *PRB1*. The CRISPR plasmids were removed by subsequent cultivation in liquid YPD confirmed by absence of growth on solid media with G418. All the primers are presented in Additional file 1: Table S1 and repair fragments for CRISPR in Additional file 1: Table S2. The oligonucleotides were aligned by heating the two corresponding oligonucleotides to 98 degrees for 5 min in equimolar amounts and let to mixture cooldown to room temperature. *E. coli* DH5α was used for plasmid amplification. The transformation protocol used for *E.coli* was according to a known protocol [52]. The strains and plasmids used and constructed in this study are listed in Tables 2 and 3.

**Table 2:** Yeast strains used in this study

Strain	Genotype	References
AAC	CEN.PK113-7D (*MAT*a *URA3 HIS3 LEU2 TRP1 MAL2-8*^*c*^* SUC2 tpi1*(41-707)::*IoxP-IoxP*	[53]
B184k	UV-mutant of AACk (*tpi1*(41–707)::*loxP*-*kanMX4-loxP*)	[19]
B184	UV-mutant of AACk (*tpi1*(41–707)::*IoxP*-*IoxP*)	This study
B184 *pep4*Δ	B184 *pep4*Δ	This study
B184 *prc1*Δ	B184 *prc1*Δ	This study
B184 *prb1*Δ	B184 *prb1*Δ	This study
B184 *pep4*Δ *prc1*Δ	B184 *pep4*Δ *prc1*Δ	This study
B184 *pep4*Δ *prb1*Δ	B184 *pep4*Δ *prb1*Δ	This study

**Table 3:** Plasmids used in this study

Plasmid	Description	References
pECAS9-gRNA-kanMX-tHFD1	2μ vector with *kanMX* marker expressing eCas9 under the *TEF1* promoter and *CYC1* terminator and the gRNA targeting *HFD1* under the *SNR52* promoter	[51]
pECAS9-gRNA-kanMX-tPEP4	2μ vector with *kanMX* marker expressing eCas9 under the *TEF1* promoter and *CYC1* terminator and the gRNA targeting *PEP4* under the *SNR52* promoter	This study
pECAS9-gRNA-kanMX-tPRC1	2μ vector with *kanMX* marker expressing eCas9 under the *TEF1* promoter and *CYC1* terminator and the gRNA targeting *PRC1* under the *SNR52* promoter	This study
pECAS9-gRNA-kanMX-tPRB1	2μ vector with *kanMX* marker expressing eCas9 under the *TEF1* promoter and *CYC1* terminator and the gRNA targeting *PRB1* under the *SNR52* promoter	This study
pNatAmyCPOT	2μ vector with cassette expressing *POT1* gene from *S. pombe* and an expression cassette with α-leader sequence and α-amylase gene under native *TPI1* promoter and terminator	[21]
pCPOT	2μ vector with cassette expressing *POT1* gene from *S. pombe* and an expression cassette with native *TPI1* promoter and terminator without recombinant protein gene	[21]
pNatZACPOT	2μ vector with cassette expressing *POT1* gene from *S. pombe* and an expression cassette with α-leader sequence and Z_HER3_1_-ABD gene under native *TPI1* promoter and terminator	This study
pNatZZACPOT	2μ vector with cassette expressing *POT1* gene from *S. pombe* and an expression cassette with α-leader sequence and Z_HER3_1_-Z_HER3_1_-ABD gene under native *TPI1* promoter and terminator	This study
pNatZAZCPOT	2μ vector with cassette expressing *POT1* gene from *S. pombe* and an expression cassette with α-leader sequence and Z_HER3_1_-ABD-Z_HER3_1_ gene under native *TPI1* promoter and terminator	This study

### Media and culture conditions

The media used for *S. cerevisiae* strain construction were YPD, YPGal, YPE and YPEG. The experiments were always performed at 30 °C and for liquid cultures at 220 rpm. YPD medium contained 10 g/L yeast extract, 20 g/L peptone, and 20 g/L glucose and was used for regular cultures. YPGal media contained 10 g/L yeast extract, 20 g/L peptone, and 20 g/L galactose and was used for induction of the Cre recombinase gene on pSH66. For the selection of the *kanMX* marker on the CRISPR plasmid, 200 mg/L G418 was added to the YPD medium. For the selection of pSH66-containing cells, 100 mg/L nourseothricin sulfate was added to YPD and YPGal medium. The YPE medium contained 10 g/L yeast extract, 20 g/L peptone, 20 g/L absolute ethanol and was solely used as a solid medium. For liquid cultivations, 30 g/L glycerol was added, and the medium referred to as YPEG. Both YPE and YPEG were only used for *S. cerevisiae* strains without CPOT plasmids since those are unable to ferment glucose as the sole carbon source [54]. To solidify the media 20 g/L agar (Merck Millipore) was added. The protein expression and physiological experiments were performed in SD2xSCAA media at 30 °C and 220 rpm. SD2xSCAA medium contained 20 g/L glucose, 6.9 g/L yeast nitrogen base without amino acids, 190 mg/L Arg, 400 mg/L Asp, 1260 mg/L Glu, 130 mg/L Gly, 140 mg/L His, 290 mg/L Ile, 400 mg/L Leu, 440 mg/L Lys, 108 mg/L Met, 200 mg/L Phe, 220 mg/L Thr, 40 mg/L Trp, 52 mg/L Tyr, 380 mg/L Val, 1 g/L BSA, 5.4 g/L Na_2_HPO_4_, and 8.56 g/L NaH_2_PO_4_·H_2_O with a pH of 6.4. Cells for protein production experiments were grown at 30 °C at 220 rpm in aerated 24-well plates CR1224 (Bioscreen) with a volume of 2.5 mL and a start OD_600_ of 0.1 or in 14 mL-cultivation tubes with a volume of 2 mL. For the binding assay experiment, a volume of 200 mL of SD2xSCAA, inoculated with an overnight culture to an OD of 0.1, was cultivated for 96 h at 30 °C at 220 rpm. The supernatant was stored at − 80 °C before being sent to Affibody AB on dry ice. The *E. coli* cells were grown in Luria–Bertani (LB) media at 37 °C and 200 rpm. Selection media contained 80 mg/L ampicillin.

### Molecular biology techniques

*Saccharomyces cerevisiae* strains were transformed using the LiAc/SS carrier method [55]. One µg of DNA was used for the transformation of plasmids and an additional 1 to 2 μg repair fragment if required. To verify deletions or test for the presence of the CPOT plasmids, colony PCR using SapphireAmp fast PCR mix (TaKaRa Bio) was performed. For DNA construction, Phusion High Fidelity DNA polymerase (Thermo Scientific) or Herculase II Fusion DNA polymerase (Aligent) was used. Restriction digestion was done using FastDigest (Thermo Scientific) products. All techniques were used according to the manufacturers' protocols unless stated otherwise.

### Growth profiler experiments

Three independent transformation colonies per strain were grown for 24 h in 1 mL SD2xSCAA media in a 14 mL-cultivation tube. Those precultures were used to inoculate the main cultures of the growth experiment in technical triplicates with a starting OD of 0.01. The *S. cerevisiae* strains were cultivated for 96 h in 250 μL SD2xSCAA media at 30 °C and 1200 rpm in 96-well plates (Enzyscreen CR1496d). The growth curves were measured using a Growth Profiler 960 (Enzyscreen).

### Protease activity experiments

For the incubation experiments, supernatants of cultivations were used. The initial cultivation was a 24 h-cultivation, if not mentioned otherwise, of a single colony in 2 mL of SD2xSCAA media. After the incubation, the culture was harvested by centrifugation at 6000 rpm for 3 min. The supernatant was kept on ice or frozen at − 20 °C. The incubation was performed in 1.5-mL Eppendorf tubes with 200 μL volume of supernatant. As positive controls, standards of purified Z_HER3_1_-ABD (1.97 mg/mL), Z_HER3_1_-Z_HER3_1_-ABD (0.77 mg/mL) and Z_HER3_1_-ABD-Z_HER3_1_ (1.34 mg/mL) provided by Affibody AB were used with a concentration of 0.01 g/L or stated otherwise. The experiments with the protease inhibitors were done by adding the protease inhibitor before adding the Affibody standard at the concentration suggested by the supplier. We used the Halt Protease Inhibitor Cocktail EDTA-free (100x) (Thermo Fisher) and 4-(2-aminoethyl) benzenesulfonyl fluoride hydrochloride (AEBSF) (final concentration 1 mM), aprotinin (final concentration 6.5 μg/mL), bestatin hydrochloride (final concentration 50 µM), leupeptin (final concentration 20 µM), E-64 (final concentration 15 µM), and pepstatin A (final concentration 10 µM). All these chemicals were purchased from Merck.

### SDS-PAGE and western blotting

The samples and controls were loaded and separated with reducing SDS-PAGE. Stain free 4–20% gels were used (Bio-rad). The proteins were transferred to 0.45-micron PVDF membranes (Bio-rad) using the Trans-Blot Turbo transfer system (Bio-rad). The blot was blocked using the Western blocker solution (Sigma Aldrich) and incubated in with either anti-Z-domain (2.87 mg/mL) (1:1000) or anti-ABD (1 mg/mL) (3:1000) antibodies, both obtained from Affibody AB followed by incubation with either anti-mouse antibody (1:5000) for anti-Z-domain or anti-rabbit (1:5000) antibody for anti-ABD antibodies, respectively. Both secondary antibodies were HRP-conjugated and visualized by using West Pico Plus HRP substrate (Thermo Fischer) and measured with a ChemidoC XRS image analyzer (Bio-Rad). As positive controls, standards of purified Z_HER3_1_-ABD (1.97 mg/mL), Z_HER3_1_-Z_HER3_1_-ABD (0.77 mg/mL) and Z_HER3_1_-ABD-Z_HER3_1_ (1.34 mg/mL) provided by Affibody AB were used with a concentration of 0.01 g/L or stated otherwise.

### Mass spectrometry analysis

Intact mass analysis was performed on an Agilent 1200 LC system equipped with a C8 RP-HPLC column (Poroshell 300SB-C8, 5 μm, 2.1 × 75 mm, Agilent) and coupled to a high-resolution Q-TOF (Bruker Maxis Impact). Buffer A was 0.1% formic acid in 10% acetonitrile, buffer B was 0.1% formic acid in 90% acetonitrile and the column temperature was 80 °C. Proteins were eluted from the LC column by using a linear gradient of solvent B from 5 to 75% over 5 min at a flow rate of 0.3 mL/min. The MS analysis was performed with an ESI source and in positive mode. MS was set in a MS only mode. The following MS parameters were used: mass range 500–4500, capillary 4500 V, nebulizer 1.2 Bar, drying gas 8.0 L/min and temperature 200 °C. The MS data were deconvoluted in Compass DataAnalysis (Bruker, version 4.4). The compound spectra were first smoothed using the Savitzky Golay algorithm with a smoothing width of 0.2 Da and 3 cycles. Then the spectrum baseline was subtracted with 0.8 flatness. Afterwards, the raw spectra were deconvoluted using the MaxEnt algorithm. For the native proteins, the spectrums were deconvoluted within the mass range of 1000–25,000 and a resolving power of 1250. The observed masses were compared to theoretical masses of different fragment sequences with GPMAW version 12.50 with a precision in Da at 0.5. Only fragment sequences with a mass accuracy ≤ 2 Da were reported.

### Binding kinetics analysis

Surface plasmon resonance (SPR) analysis by Biacore 8 K (Cytiva, Marlborough, MA) was performed at 25 °C in a run buffer of HBS-EP (0.01 M HEPES pH 7.4, 0.15 M NaCl, 3 mM EDTA, 0.005% v/v Surfactant P20, Cytiva) and with 15 mM HCl as regeneration solution. Recombinant human ErbB3/Her3 Fc (R&D Systems, Minneapolis, MN) was immobilized by standard amine coupling (1-ethyl-3-(3-dimethylaminopropyl)carbodiimide (EDC) and N-hydroxysuccinimide (NHS)) on a CM5 sensor chip (Cytiva, Marlborough, MA) at ~ 1000 RU. The coated chip was preconditioned by three regeneration rounds to stabilize surfaces prior to injection of analyte. Binding of Affibody molecules to HER3 was analyzed by single cycle kinetics via injection of analyte at five different concentrations of purified Affibody molecule (1.25, 2.5, 5, 10 and 20 nM) over immobilized HER3/Fc. The experiment was performed in duplicates. Biacore Insight Evaluation Software was used to process, analyze and fit data.

### Fed-batch bioreactor cultivations

For the Bioreactor experiments, the Na_2_HPO_4_ and NaH_2_PO_4_•2H_2_O were replaced by KH_2_PO_4_ with a concentration of 2 g/L in the SD2xSCAA media. The batch fermentations were performed in 1-L bioreactors (DasGip) with a start OD_600_ of 0.01 in 500 mL of SD2xSCAA media. The conditions in the reactors were controlled and maintained at a pH of 6.0 by 4 M KOH, agitation at 600 rpm, temperature at 30 °C degrees and airflow of 30 L/h. The batch experiments were performed in biological quadruplicates. After all the glucose was consumed, culture broth was removed until a start volume of 250 mL. The conditions of the fed-batch were set to a pH of 6.0, maintained by 4 M KOH and 3 M HCl, agitation was set to 600 rpm, the temperature was set to 30 °C degrees, a constant airflow of 18 L/h and an exponentially increasing feed rate of 0.05 h^−1^. When the dissolved oxygen in the bioreactor would decrease below 30% the airflow and agitation would increase. The airflow would increase to 48 L/h and agitation would increase to 1000 rpm. When both the agitation and airflow were at their maximum levels the feeding was changed to a pulse feed controlled the by dissolved oxygen level. The feed would stop if the dissolved oxygen was below 25% which resulted in a pulsed feeding rate around 8 mL/h. Two types of media were used for the fed-batch experiments. The first medium was the low-glucose feed with 200 g/L, 69 g/L yeast nitrogen base without amino acids, 50 g/L casamino acids (Formedium), 1 g/L BSA, and 20 g/L KH_2_PO_4._ After adding an average of 320 mL of the low glucose feed the low-glucose feed was replaced by a high-glucose feed with the same composition as the low-glucose feed except for a glucose concentration of 400 g/L. The fermentation was ended after addition of 230 mL of the high-glucose feed. The fed-batch experiments were conducted in biological triplicates.

### Affibody molecule quantification by Bio-Layer Interferometry (BLI)

The broth samples were centrifugated for 20 min at 4000 rpm to remove the biomass and the supernatant was stored at − 80 °C. After thawing some precipitation appeared in the samples. To redissolve the precipitation the samples were 1:1 diluted in 1 NaCl in two duplicates per fermenter per sample point and transported on dry ice. Upon arrival the sample were thawed and either not diluted or diluted 1:3.75, 1:7.5 in sample diluent (1 × PBS, 0.1% BSA, 0.02% Tween 20) based on the expected concentration. 225 µl of the pre-dilution was mixed with either 75 µL sample diluent (unspiked) or 75 µl of 100 µg/mL Z_HER3_1_-ABD-Z_HER3_1_ (produced by *E. coli*) (spiked) was added resulting in a 1:5, 1:10 or 1:1.33 final dilution. 200 µL of the spiked and unspiked sample was moved to a black flat-bottomed 96-well plate (Greiner, #655209) together with 200 µL of the samples for the standard curve, 200 µL sample diluent (reference well) and 200 µL cultivation medium (negative control). High precision streptavidin (SAX) biosensors (ForteBio, #18–0037) which were previously loaded offline (Technical Note #10, ForteBio) with 50 µg/mL biotinylated HSA (Recombumin Prime, Albumedix; Thermo Fisher #A39259) (MCR 1:1) were placed for 10 min in sample diluent before the measurements. The data was acquired using an Octet HTX system and Octet Data Acquisition software (ForteBio, ver. 12). The measurement settings were as follows: 8 channel read head, 5.0 Hz acquisition rate, quantitation step: 120 s, 400 rpm, plate temperature 30 °C. The quantity was determined based on the six-point standard curve with Z_HER3_1_-ABD-Z_HER3_1_ produced by *E. coli* (100 µg/mL-3.13 µg/mL). Data Analysis HT (ForteBio, ver. 12) was used to process the data. The reference well signal was subtracted from the samples and standard curve, and results with 70–143% spike recovery were included in the calculations. The assay was run twice for each sample.

## Supplementary Information

**Additional file 1: Figure S1.** SDS PAGE (cropped) of degradation experiment with AAC and B184k. **Figure S2.** Binding assay kinetics of Z_HER3_1_ -ABD-Z_HER3_1_. **Figure S3.** Growth profiles of B184 and B184 *pep4Δ* while producing Z_HER3_1_ -ABD-Z_HER3_1_. **Figure S4.** Semi-log plot of cell dry weight measurements during batch fermentation. **Figure S5.** Western blot against the ABD (cropped) of Z_HER3_1_ -ABD-Z_HER3_1_ produced by B184, B184 *pep4Δ* and B184 *pep4Δprc1Δ.*.** Table S1.** Primers used in this study.** Table S2.** Repair fragments for CRISPR used in this study.

## Data Availability

Materials are available from the corresponding author upon reasonable request.
