# Supplementary material for: Engineering Saccharomyces cerevisiae for the production and secretion of Affibody molecules
Source: Microb Cell Fact. 2022 Mar 9;21:36. doi: 10.1186/s12934-022-01761-0 (PMC8905840; doi:10.1186/s12934-022-01761-0)
Supplement: Supplementary file 1 — Additional file 1: Figure S1. SDS PAGE (cropped) of degradation experiment with AAC and B184k. Figure S2. Binding assay kinetics of ZHER3_1 -ABD-ZHER3_1. Figure S3. Growth profiles of B184 and B184 pep4Δ while producing ZHER3_1 -ABD-ZHER3_1. Figure S4. Semi-log plot of cell dry weight measurements during batch fermentation. Figure S5. Western blot against the ABD (cropped) of ZHER3_1 -ABD-ZHER3_1 produced by B184, B184 pep4Δ and B184 pep4Δprc1Δ.. Table S1. Primers used in this study. Table S2. Repair fragments for CRISPR used in this study. [file 12934_2022_1761_MOESM1_ESM.pdf]

Supplementary material

Engineering *Saccharomyces cerevisiae* for the production and secretion of Affibody molecules.

Veronica Gast, Anna Sandegren, Finn Dunås, Siri Ekblad, Rezan Güler, Staffan Thorén, Marta Tous Mohedano, Mikael Molin, Martin K M Engqvist and Verena Siewers.

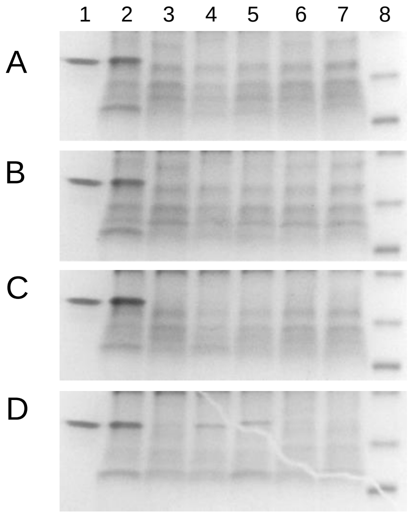

Supplementary Figure 1. SDS-page of Affibody molecule standards after incubation in spent supernatant from several timepoints during a cultivation. SDS-PAGE of spent supernatant harvested after 1 to 4 days, after overnight incubation with Z_{HER3_1}-ABD-Z_{HER3_1} standards. (A) spent supernatant of AAC producing α-amylase, (B) spent supernatant of B184k producing α-amylase (C) spent supernatant of AAC not expressing a recombinant protein and (D) spent supernatant of B184k not expressing a recombinant protein. Lane 1 contains the control of the Affibody molecule standard in water, Lane 2 contains the positive control with the spent supernatant after 72 h cultivation with the Affibody molecule standard and protease inhibitor cocktail, the cocktail contains a component that results in the lower thick band in the lane, lane 3 contains the spent supernatant after 72 h cultivation without Affibody molecule standard, lane 4 contains the spent supernatant after 24 h cultivation with Affibody molecule standard, lane 5 contains the spent supernatant after 48 h cultivation with Affibody molecule standard, lane 6 contains the spent supernatant after 72 h cultivation with Affibody molecule standard, lane 7 contains the spent supernatant after 96 h cultivation with Affibody molecule standard and lane 8 contains a protein ladder.

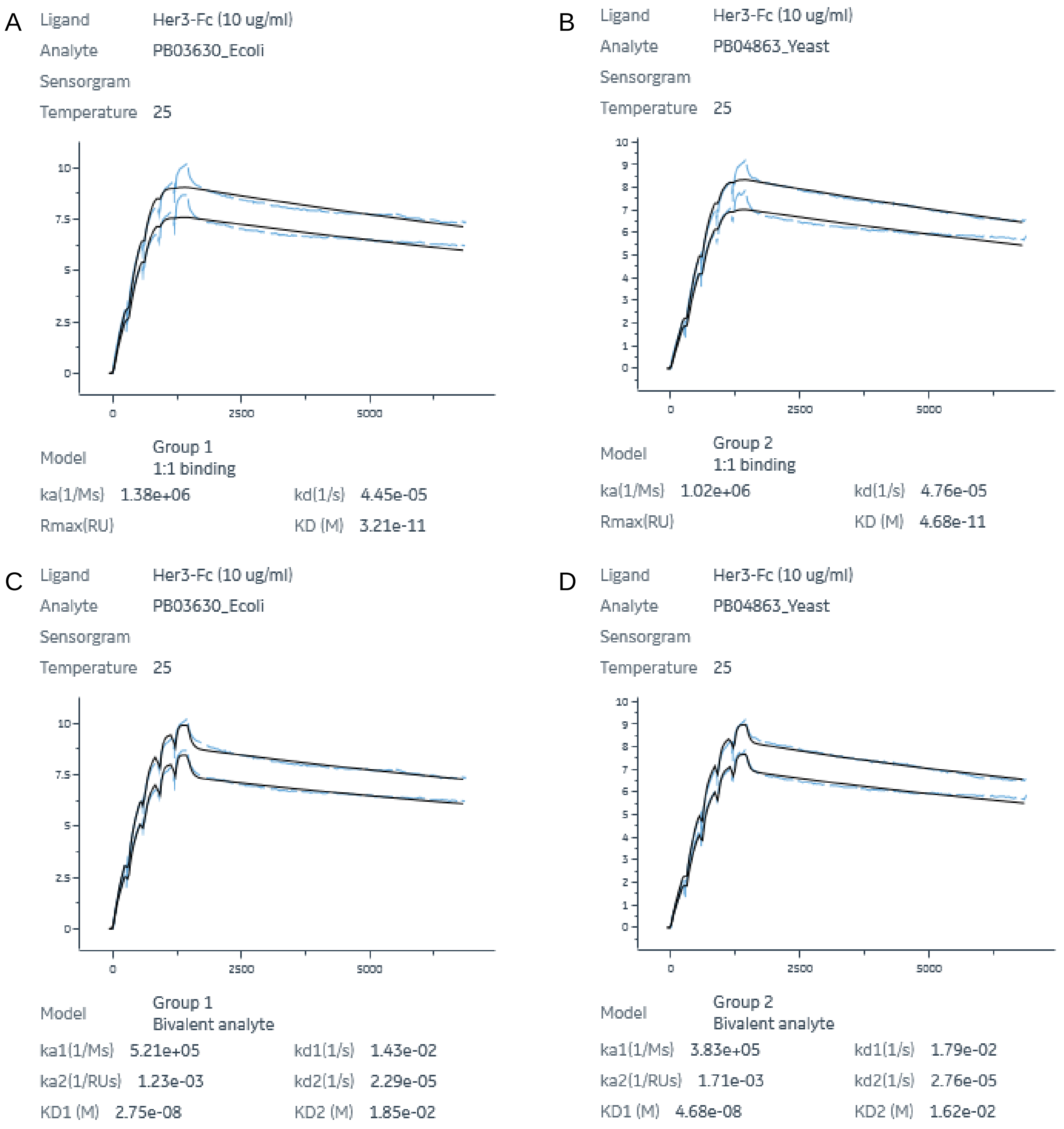

Supplementary Figure 2. Binding assay kinetics of Z_{HER3_1} -ABD- Z_{HER3_1}. Results of the binding assay of the produced Z_{HER3_1} -ABD- Z_{HER3_1}. (A, B) Interaction fits of Z_{HER3_1} -ABD- Z_{HER3_1} produced by *E. coli* (A) and *S. cerevisiae* (B). (C, D) Bivalent analyte interaction fits of Z_{HER3_1} -ABD- Z_{HER3_1} produced by *E. coli* (C) and *S. cerevisiae* (D). The experiments were performed in duplicates.

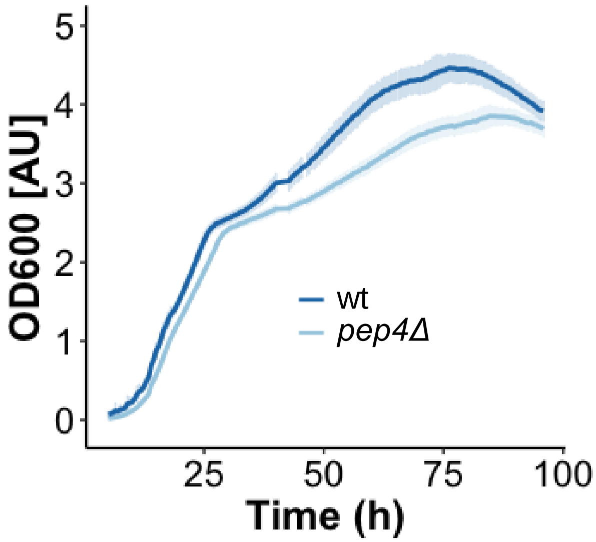

Supplementary Figure 3. Growth profiles of B184 and B184 *pep4Δ* while producing Z_{HER3_1}-ABD- Z_{HER3_1}. OD_{600} data from 96 h of growth in SD2xSCAA media of B184 and B184 *pep4Δ* expressing Z_{HER3_1}-ABD- Z_{HER3_1}. The graph shows averaged data based on biological triplicates with technical triplicates. The lighter bars show the standard deviation. The first 5 h were excluded from the graphs due to noise.

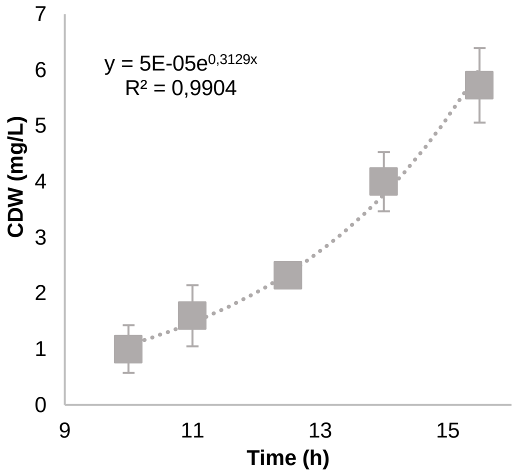

Supplementary Figure 4. Semi-log plot of cell dry weight measurements during the batch phase. The errors bars are based on the standart deviations of the quaduplicates. The averages are fitted to an exponential curve.

Z_{HER3_1}-ABD-Z_{HER3_1}

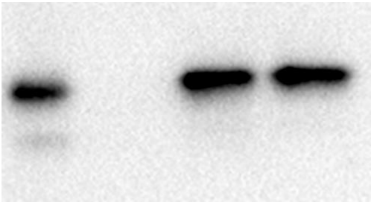

Supplementary Figure 5. Western blot against the ABD of Z_{HER3_1}-ABD-Z_{HER3_1} produced by B184, B184 *pep4Δ* and B184 *pep4Δprc1Δ*. Western blot from a reducing SDS-PAGE. The membrane was blotted with anti-ABD (1:1000) antibody followed by an anti-Rabbit (1:5000) secondary antibody. SDS-PAGE separated the supernatant after 96 h of cultivation of B184, B184 *pep4Δ* and B184 *pep4Δprc1Δ* producing Z_{HER3_1}-ABD-Z_{HER3_1}. The lane marked with st contains Z_{HER3_1}-ABD-Z_{HER3_1} standard in water (0.05 g/L).

Supplementary table 1. Primers used in this study

#	Alignment	Template	Overhang/explanation	Sequence
1	Fw	Z_{HER3_1}-ABD	α-leader CPOT	CTTTGGATAAAAGAGAAGAAGGTGA AGGATCCATGGCTGAAGC
2	Rv	Z_{HER3_1}-ABD	*TPI1* terminator CPOT	TATTAATCTTAGTTTCTAGACTCGAG TTATGGTAATGCAGC
3	Fw	Z_{HER3_1}-Z_{HER3_1}-ABD	α-leader CPOT	CTTTGGATAAAAGAGAAGAAGGTGA AGGATCCATGGCAGAAGC
4	Rv	Z_{HER3_1}-Z_{HER3_1}-ABD	*TPI1* terminator CPOT	TATTAATCTTAGTTTCTAGACTCGA GTTAAGGTAGAGCTGCC
5	Fw	Z_{HER3_1}-ABD-Z_{HER3_1}	α-leader CPOT	CTTTGGATAAAAGAGAAGAAGGTG AAGGATCCATGGCTGAGGC
6	Rv	Z_{HER3_1}-ABD-Z_{HER3_1}	*TPI1* terminator CPOT	TATTAATCTTAGTTTCTAGACTCGA GTTATTTGGGAGCTTG
7	Fw	α-leader CPOT	Verification CPOT	GCTCAAATTCCAGCTGAAGC
8	Fw	pECAS9-gRNA-kanMX-t	*PEP4* gRNA	ATAAATGATCCCCTAGAAAAAACAACTTCGG TTTTAGAGCTAGAAATAGCAAG
9	Rv	pECAS9-gRNA-kanMX-t	*PEP4* gRNA	GCTCTAAAACCGAAGTTGTTTTTTCTAGGGG ATCATTTATCTTTCACTGCG
10	Fw	pECAS9-gRNA-kanMX-t	*PRC1* gRNA	ATAAATGATCGTTATAGACATCCTTACCAGG TTTTAGAGCTAGAAATAGCAAG
11	Rv	pECAS9-gRNA-kanMX-t	*PRC1* gRNA	GCTCTAAAACCTGGTAAGGATGTCTATAACG ATCATTTATCTTTCACTGCG
12	Fw	pECAS9-gRNA-kanMX-t	*PRB1* gRNA	ATAAATGATCCTTAACGACGAAGATCTCGAGT TT TAGAGCTAGAAATAGCAAG
13	Rv	pECAS9-gRNA-kanMX-t	*PRB1* gRNA	GCTCTAAAACTCGAGATCTTCGTCGTTAAGGA TCA TTTATCTTTCACTGCG
14	Fw	pECAS9-gRNA-kanMX-t	Construct 'left' fragment for pECAS9-gRNA-kanMX with new gRNA	GGAACAACACAAACACTAC
15	Rv	pECAS9-gRNA-kanMX-t	Construct 'right' fragment for pECAS9-gRNA-kanMX with new gRNA	CAAAGGAAATGATAGCATTGAA
16	Fw	pECAS9-gRNA-kanMX-t	Verification pECAS9-gRNA-kanMX	GGACGCTCGAAGGCTTTAAT
17	Fw	Chromosomal DNA	Verification deletion *PEP4* outside *PEP4* gene	GGACGCTCGAAGGCTTTAAT
18	Fw	Chromosomal DNA	Verification deletion *PEP4* inside *PEP4* gene	CAGCAGCATAGAACAATGG
19	Rv	Chromosomal DNA	Verification deletion *PEP4* outside *PEP4* gene	CCGCCATTTTCAGTATCC
20	Fw	Chromosomal DNA	Verification deletion *PRC1* outside *PRC1* gene	GCCTAGTGACCTAGTATTTAATCC
21	Fw	Chromosomal DNA	Verification deletion *PRC1* inside *PRC1* gene	GCCAGCAAAAAAGCTCC
22	Rv	Chromosomal DNA	Verification deletion *PRC1* outside PRC1 gene	GCTGTTCTTTGAATTAGGACC
23	Fw	Chromosomal DNA	Verification deletion *PRB1* outside *PRB1* gene	GGAGGTCTTTTTTGATGTGC
24	Fw	Chromosomal DNA	Verification deletion *PRB1* inside *PRB1* gene	CATTTGATCAGATAAGTGATCTGC
25	Rv	Chromosomal DNA	Verification deletion *PRB1* outside *PRB1* gene	TCTTGGGCTTCTTTTTGG

Supplementary table 2. Repair oligos for CRISPR used in this study

Repair oligo	Sequence
Repair oligo for *PEP4* deletion (1/2)	CTTATAAAAGCTCTCTAGATGGCAGAAAAGGATAGGGCGGA GAAGTAAGAAAAGTTTAGCGTTAGTTTTGGTTTTTGTTTGAA TTTTATTTGGATTAAATACTAGGTCACTAGGC
Repair oligo for *PEP4* deletion (2/2)	GCCTAGTGACCTAGTATTTAATCCAAATAAAATTCAAACAAAA ACCAAAACTAACGCTAAACTTTTCTTACTTCTCCGCCCTATCC TTTTCTGCCATCTAGAGAGCTTTTATAAG
Repair oligo for *PRC1* deletion (1/2)	GAGAAAGAATACTCACTAGAGATTGTTTCTTTTCTACTCAACTTA AAGTATACATACGCTAGCGTATATGTGTAGGCACACCGTTTTTA TTATCAGCTACGATCGAAATATATACGTTTT
Repair oligo for *PRC1* deletion (2/2)	AAAACGTATATATTTCGATCGTAGCTGATAATAAAAACGGTGTG CCTACACATATACGCTAGCGTATGTATACTTTAAGTTGAGTAG AAAAGAAACAATCTCTAGTGAGTATTCTTTCTC
Repair oligo for *PRB1* deletion (1/2)	CGAGACGCCTAAGGAAAGAAAAAGAAAAAAAAAGCAGCTGAAAT TTTTCTAAATGAAGAACTTTGCTTGTTAGAATTAGGTTTAGTTTGT TTTTTTATTGGCGATGAAGCTAATTGGACT
Repair oligo for *PRB1* deletion (2/2)	AGTCCAATTAGCTTCATCGCCAATAAAAAAACAAACTAAACCTAA TTCTAACAAGCAAAGTTCTTCATTTAGAAAAATTTCAGCTGCTTT TTTTTTCTTTTTCTTTCCTTAGGCGTCTCG
